# Understanding local determinants of dengue: a geographically weighted panel regression approach in Yogyakarta, Indonesia

**DOI:** 10.1186/s41182-025-00734-4

**Published:** 2025-04-14

**Authors:** Marko Ferdian Salim, Tri Baskoro Tunggul Satoto, Danardono

**Affiliations:** 1 Doctorate Program of Medical and Health Science, Faculty of Medicine, Public Health and Nursing, Universitas Gadjah Mada, Yogyakarta, 55281 Indonesia; 2 Department of Parasitology, Faculty of Medicine, Public Health and Nursing, Universitas Gadjah Mada, Yogyakarta, 55281 Indonesia; 3 Department of Mathematics, Faculty of Mathematics and Natural Sciences, Universitas Gadjah Mada, Yogyakarta, 55281 Indonesia; 4 Department of Health Information and Services, Vocational College, Universitas Gadjah Mada, Yogyakarta, 55281 Indonesia

**Keywords:** Dengue, Local determinants, Spatiotemporal analysis, Geographically weighted panel regression, Fixed-effects model

## Abstract

**Background:**

Dengue remains a major public health concern in tropical regions, including Yogyakarta, Indonesia. Understanding its spatiotemporal patterns and determinants is crucial for effective prevention strategies. This study explores the spatiotemporal determinants of dengue incidence and evaluates the spatial variability of predictors using a geographically weighted panel regression (GWPR) approach.

**Methods:**

This ecological study applied a spatiotemporal approach, analyzing dengue incidence across 78 sub-districts in Yogyakarta from 2017 to 2022. The dataset included meteorological variables (rainfall, temperature, humidity, wind speed, and atmospheric pressure), sociodemographic data (population density), and land-use characteristics (built-up areas, crops, trees, water bodies, and flooded vegetation). A GWPR model with a Fixed Exponential kernel was used to assess local variations in predictor influence.

**Results:**

The Fixed Exponential Kernel GWPR model showed strong explanatory power (Adjusted *R*^2^ = 0.516, RSS = 43,097.96, AIC = 28,447.38). Local *R*-Square values ranged from 0.25 (low-performing sub-districts) to 0.75 (high-performing sub-districts), indicating significant spatial heterogeneity. Sub-districts such as Pakem, Cangkringan, and Girimulyo exhibited high local *R*^2^ values (>0.75), indicating robust model performance, whereas Kalibawang showed lower values (<0.25), suggesting weaker predictive power. High-dengue-burden sub-districts, including Kasihan (0.743), Banguntapan (0.731), Sewon (0.716), Wonosari (0.623), and Wates (0.540), demonstrated stronger associations between dengue incidence and key predictors. In Wonosari, the most influential predictors were Rainfall Lag 1, Rainfall Lag 3, temperature, humidity, wind speed, atmospheric pressure, and land-use variables, while in Wates, significant predictors included Rainfall Lag 1, Rainfall Lag 3, atmospheric pressure, and land-use factors. Lower model performance in Sedayu and Kalibawang suggests the necessity of incorporating additional predictors such as sanitation conditions and vector control activities.

**Conclusions:**

The GWPR model provides valuable insights into the spatiotemporal dynamics of dengue incidence, emphasizing the role of localized predictors. Spatially adaptive prevention strategies focusing on high-risk areas are essential for effective dengue control in Yogyakarta and similar tropical regions.

**Supplementary Information:**

The online version contains supplementary material available at 10.1186/s41182-025-00734-4.

## Introduction

Dengue fever is an infectious disease that poses a significant public health challenge in tropical and subtropical regions, particularly in Indonesia [1]. The transmission of the dengue virus by Aedes aegypti and Aedes albopictus mosquitoes has led to a global surge in cases, with an estimated 390 million infections per year, of which approximately 96 million cases exhibit clinical manifestations [2]. Southeast Asia, including Indonesia, is the region with the highest dengue burden, where environmental factors, population density, and climate change contribute to the increased transmission of the dengue virus [2–5].

In Indonesia, dengue fever has been reported since 1968 and remains a public health concern, with fluctuating transmission patterns observed annually. The Special Region of Yogyakarta is classified as an endemic area, with an incidence rate (IR) of 29.9 per 100,000 population, exceeding the national standard [6]. Over the past five years, dengue cases in the Special Region of Yogyakarta have shown significant fluctuations, peaking in 2019 and 2020, with more than 3000 cases per year [7]. Various environmental, climatic, and sociodemographic factors have been linked to the rising incidence of dengue fever; however, studies that simultaneously account for spatiotemporal dynamics remain limited [8, 9]. Most predictive models, such as ARIMA, SARIMA, support vector machines (SVM), artificial neural networks (ANN), and generalized additive models (GAM), primarily focus on temporal trends, neglecting spatial heterogeneity [10–12]. However, dengue cases tend to cluster in both space and time, making spatial analysis crucial for understanding disease transmission patterns [8].

Despite increasing evidence of the impact of environmental and climatic factors on dengue transmission, existing models often fail to account for localized variations [13, 14]. Many studies have predominantly relied on global or temporal models, resulting in generalized predictions that may not be effective for region-specific interventions [15, 16]. Since dengue transmission is shaped by complex interactions between climate, environment, and human activity, there is an urgent need for adaptive approaches that incorporate these dynamic factors [17–23]. In Indonesia, where climate variability continues to rise and dengue outbreaks remain unpredictable, spatially informed risk assessments could enhance early detection and facilitate targeted control measures [24].

The GWPR approach addresses spatial and temporal heterogeneity by allowing regression coefficients to vary across geographic locations, enabling localized analysis of relationships between predictors and dengue incidence [9]. Unlike global models, GWPR provides adaptive estimations and accommodates hierarchical structures, recognizing the influence of both temporal and region-specific factors [8]. This study applies GWPR to explore the spatiotemporal determinants of dengue fever incidence and evaluate the spatial variability of predictors in the Special Region of Yogyakarta. Specifically, the model analyzes the influence of weather factors (rainfall, temperature, humidity, atmospheric pressure, wind speed), sociodemographic factors (population density), and environmental factors (built area, crops area, trees area, water area, and flooded vegetation area) on dengue transmission dynamics. The objective of this study is to gain a deeper understanding of the spatial and temporal patterns of dengue cases, providing insights into localized variability and supporting data-driven prevention and control strategies. By utilizing a GWPR-based approach, this research aims to enhance early detection of dengue risk, enabling more effective and targeted interventions to reduce morbidity and mortality in the region.

## Methods

This study was conducted in the Special Region of Yogyakarta, Indonesia, situated between 110°00′–110°50′E longitude and 7°33′–8°12′S latitude, covering an area of 3186 km^2^. This region consists of four districts (Bantul, Gunung Kidul, Kulon Progo, and Sleman) and one municipality (Yogyakarta City). With a tropical monsoon climate, the area experiences distinct wet and dry seasons, which significantly influence dengue transmission dynamics. The region’s heterogeneous landscape, comprising densely populated urban centers and geographically diverse rural areas, makes it an ideal setting for a spatiotemporal analysis of dengue incidence.

This study employed a retrospective observational analytic design within an ecological spatiotemporal framework. The analysis utilized panel data from 78 sub-districts, recorded monthly from January 2017 to December 2022. Secondary data sources included dengue surveillance reports from Primary Health Centres and the District Health Office, meteorological data from NASA’s POWER Data Access Viewer, sociodemographic data from BPS-Statistics Indonesia, and land-use data derived from Sentinel-2 satellite imagery through ESRI’s Land Cover Explorer.

To enable a comprehensive spatiotemporal analysis, this study compiled panel data integrating both spatial and temporal dimensions. The dependent variable was dengue incidence, defined as the total number of monthly dengue cases per sub-district. Independent variables included climatic factors (rainfall, including lag effects for 1, 2, and 3 months; temperature; relative humidity; wind speed; and atmospheric pressure), sociodemographic factors (population density), and environmental factors (built area, crops area, trees area, water area, and flooded vegetation area). Rainfall lag variables (Lag 1, Lag 2, and Lag 3) were included to capture the delayed effects of rainfall on mosquito breeding and dengue transmission. Lag 1 reflects initial larval development, Lag 2 represents adult mosquito emergence, and Lag 3 accounts for virus incubation. These lags improve the model’s ability to assess the correlation between rainfall and dengue incidence over time [25].

The dataset underwent a preprocessing stage, including data cleaning, normalization, temporal alignment, and spatial standardization, to ensure consistency across variables. To assess potential collinearity among predictor variables, a multicollinearity test was performed using the variance inflation factor (VIF). Variables with VIF values greater than 10 were considered indicative of multicollinearity and subject to further evaluation or removal from the model.

The GWPR method extends geographically weighted regression (GWR) by incorporating spatial and temporal dimensions, allowing for localized parameter estimation across time. Unlike global models, GWPR captures spatial heterogeneity, enabling the relationship between dengue incidence and predictor variables to vary across locations. The Fixed Exponential kernel was selected based on model evaluation criteria (RSS, Adjusted *R*^2^, AIC, and AICc), demonstrating superior performance over other kernels. GWPR was also chosen over machine learning-based spatial models (e.g., spatial random forests, deep learning) due to its interpretability and ability to explicitly model spatially varying relationships. While machine learning methods excel in predictive accuracy, they lack explainability in how risk factors influence dengue incidence across different locations. Given the study’s focus on understanding localized determinants rather than just prediction, GWPR provides a more effective approach to identify spatiotemporal patterns, offering valuable insights for targeted dengue control strategies.

Kernel functions in GWPR define the spatial weighting scheme based on the distance between observations. The most used kernel functions include Gaussian, Bisquare, and Exponential, each offering distinct weighting characteristics. The Gaussian kernel applies a continuously decreasing weight function, whereas the Bisquare kernel assigns weights that sharply drop beyond a certain distance threshold. The Exponential kernel, on the other hand, allows for a gradual decrease in weights without an absolute cutoff, making it particularly useful for capturing smooth spatial variations. Furthermore, bandwidth selection is crucial in GWPR, with two main approaches: fixed bandwidth and adaptive bandwidth. A fixed bandwidth maintains a consistent spatial extent for weighting observations, which is suitable for datasets with uniform spatial distributions. In contrast, an adaptive bandwidth adjusts the range dynamically based on data density, making it more appropriate for highly variable spatial structures. The choice of fixed or adaptive bandwidth impacts the model’s sensitivity to local variations, affecting how well spatial patterns in dengue incidence are captured [26–28].

Given the study’s objective—to identify localized determinants of dengue incidence rather than solely optimizing predictive accuracy—GWPR was deemed the most appropriate method. Statistical analyses were conducted in R Studio using the “GWmodel” package for GWPR.

## Results

### Descriptive statistic

This study recorded 13,283 dengue cases in the Special Region of Yogyakarta from 2017 to 2022, with an average incidence of 2.365 cases per sub-district per month, ranging from 0 to 82 cases. Weather conditions during the study period included an average rainfall of 210.12 mm, a temperature of 25.82 °C, relative humidity of 83.98%, wind speed of 3.54 m/s, and atmospheric pressure of 98.91 kPa. Population density varied significantly, averaging 3651 people/km^2^, with a range of 252 to 22,956 people/km^2^. Environmental factors also exhibited variation, with built areas averaging 1574.89 hectares, tree areas 1082.10 hectares, crop areas 819.60 hectares, and smaller water bodies at 27.17 hectares (Table 1).

**Table 1:** Descriptive statistics results

Variables	Sum	Mean	Min	Max	SD
Dengue cases	13,283	2.365	0	82	4.422
Weather factors					
Rainfall	1,180,014.4	210.116	0	796.29	154.327
Rainfall Lag 1	1,170,996.5	208.510	0	796.29	153.282
Rainfall Lag 2	1,168,922.1	208.141	0	796.29	152.565
Rainfall Lag 3	1,174,864.3	209.199	0	796.29	153.707
Average temperature	145,009.36	25.820	21.15	28.15	1.147
Relative humidity	471,612.51	83.976	60.25	92.12	4.630
Wind speed	19,864	3.537	1.52	5.45	0.909
Atmospheric pressure	555,462.1	98.907	93.88	100.73	1.559
Sociodemographic factors					
Population density	20,501,542	3,650.5595	252	22.956	5091.546
Environmental factors					
Built area	8,844,599.6	1,574.893	64.025	4303.079	997.912
Crops area	4,602,894.4	819.604	0	6282.976	931.104
Water area	152,569.13	27.167	0	324.137	50.070
Trees area	6,077,072.5	1082.099	0	9069.382	1665.042
Flooded vegetation area	4236.33	0.754	0	47.9640	3.529

**Fig. 1:**
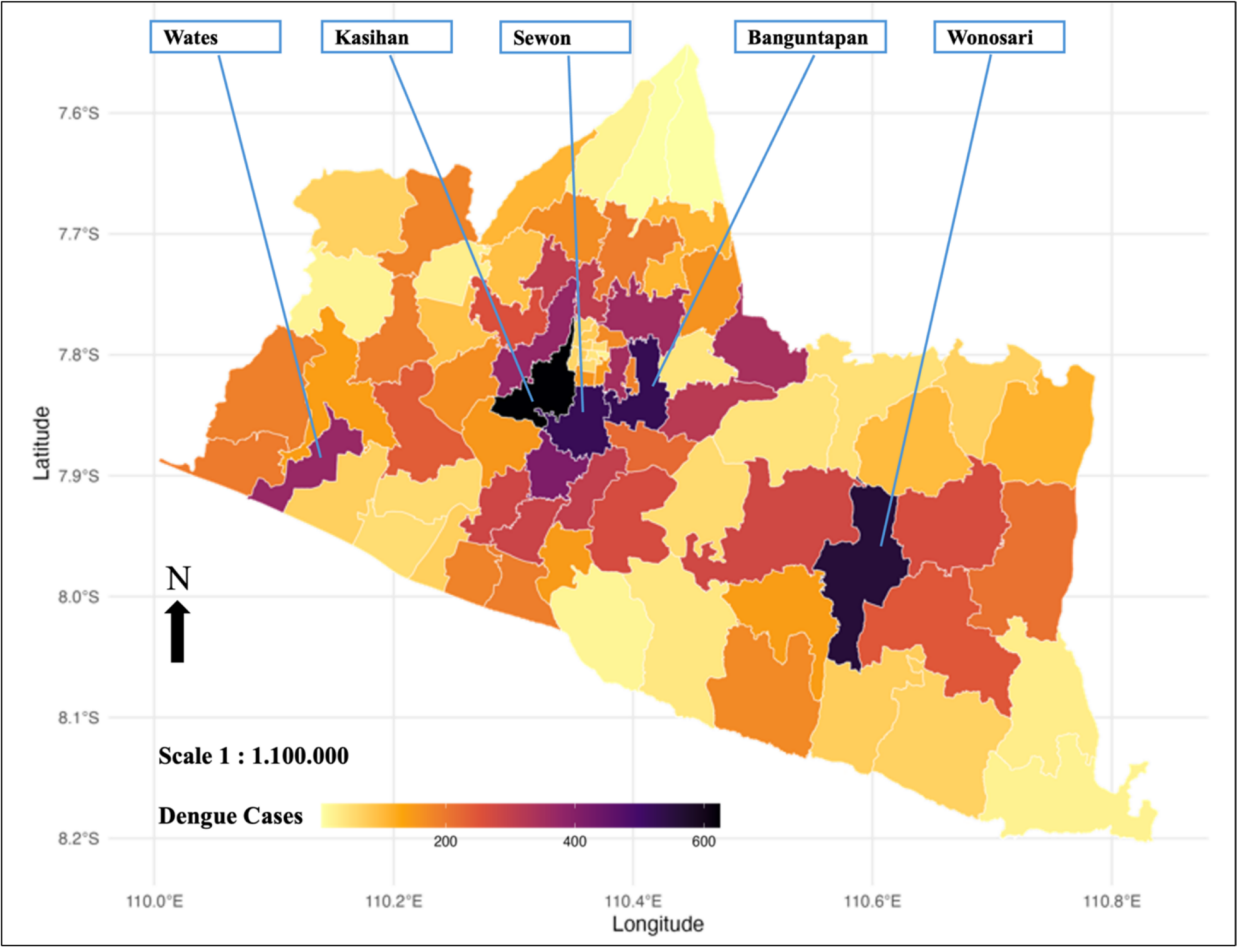
Spatial distribution of total dengue incidence in 2017–2022

Figure 1 illustrates the spatial distribution of dengue cases, which demonstrates significant variation across sub-districts. The highest concentrations are observed in central areas such as Kasihan, Banguntapan, and Sewon, characterized by high population densities and better accessibility. Conversely, regions like Wonosari in the east and Wates in the west highlight the influence of local factors, including climate and land use, on case numbers.

## Analysis of geographically weighted panel regression (GWPR)

The GWPR analysis was conducted to examine the spatial and temporal influences on dengue incidence in the Special Region of Yogyakarta. This approach allows for the identification of local variations in the relationships between dengue cases and climatic (rainfall, temperature, humidity, wind speed, atmospheric pressure), sociodemographic (population density), and environmental factors (land-use types). Using panel data from 78 sub-districts over 72 months (5616 observations), this study initially employed classical panel data regression to determine the most suitable model. The multicollinearity test (Table S1) confirmed that all predictor variables had VIF values below 10, indicating no significant collinearity issues. Therefore, all selected predictors were retained in the model for further analysis. Model selection tests (Table S2), including the Chow Test, Hausman Test, and Breusch–Pagan LM Test, were performed to compare the Common-Effect Model (CEM), Fixed-Effect Model (FEM), and Random-Effect Model (REM).

The results indicated that the FEM is the most appropriate model, as it effectively captures spatial and temporal variations. The Chow Test confirmed FEM’s superiority over CEM (*p*-value < 0.0001), while the Hausman Test further supported FEM over REM (*p*-value < 0.0001), emphasizing its ability to account for fixed individual effects across sub-districts. The Breusch–Pagan LM Test showed that REM is preferable to CEM, reinforcing the inadequacy of CEM for this dataset. These findings highlight the importance of FEM in analyzing dengue dynamics and provide a foundation for advanced spatial modeling through GWPR to better understand the localized impact of predictive factors on dengue transmission.

The statistical tests (Table S3) revealed violations of normality, autocorrelation, and heteroskedasticity in the fixed-effects model (FEM), indicating its limitations in capturing dengue transmission dynamics. While FEM was selected as the best classical panel data regression model (based on Chow, Hausman, and Breusch–Pagan LM Tests), its assumptions were significantly violated (*p* < 0.0001), suggesting that a global model is insufficient for addressing spatial heterogeneity. To overcome this, GWPR was chosen as it allows for spatially varying coefficients, providing a more accurate representation of localized dengue determinants.

Given these limitations, a spatiotemporal analytical method like GWPR is more appropriate, as it allows for localized parameter estimation, capturing spatial and temporal heterogeneity in dengue transmission patterns. Unlike traditional panel regression models, GWPR does not require the assumption of normally distributed residuals, as it accommodates spatial non-stationarity and localized variations. Moreover, GWPR inherently accounts for spatial dependencies, reducing the impact of autocorrelation by allowing model parameters to vary across different locations. The adoption of GWPR, therefore, provides a more nuanced and region-specific analysis, making it better suited for understanding the localized determinants of dengue incidence.

The selection of the Fixed Exponential Kernel in GWPR was based on model performance metrics (Table S4). This kernel achieved the lowest RSS (43,097.96), highest *R*^2^ (0.607), and lowest AIC (28,447.38), indicating strong model fit and predictive accuracy. The Fixed Kernel was chosen over the Adaptive Kernel to maintain bandwidth consistency across urban and rural areas, ensuring uniform spatial smoothing. The Exponential function was preferred over Bisquare and Gaussian as it assigns higher weights to nearby observations and gradually decreases influence over distance, effectively capturing localized variations in dengue incidence. Based on these results, the Fixed Exponential Kernel was identified as the most suitable approach for analyzing spatiotemporal determinants of dengue transmission in Yogyakarta.

The Fixed Exponential kernel was identified as the optimal model for GWPR analysis, demonstrating superior performance with the lowest Residual Sum of Squares (RSS = 43,097.96) and the highest *R*^2^ (0.607) and Adjusted *R*^2^ (0.516), indicating strong explanatory power. Additionally, it achieved the lowest AIC (28,447.38) and AICc (30,015.01), ensuring an optimal balance between accuracy and complexity. Based on these results (Table S4), the Fixed Exponential kernel was selected for analyzing spatiotemporal factors influencing dengue incidence.

The GWPR analysis using a Fixed Exponential kernel (Table 2) revealed significant spatial variability in the influence of predictors on dengue incidence across sub-districts. Climatic factors (rainfall, temperature, humidity, and atmospheric pressure) had dominant effects in most areas, while environmental factors (built areas, trees, and flooded vegetation) showed localized impacts. The coefficient distribution (Min, Q1, Median, Q3, Max) highlights variations in predictor influence across locations. Median values are used for interpretation, ensuring a stable representation of effects across sub-districts. Rainfall, temperature, and humidity generally exhibited positive associations with dengue cases, while land-use variables showed heterogeneous effects.

**Table 2:** Model coefficient statistics for GWPR with exponential kernel in predicting dengue cases

Variable	Minimum	Quartile 1	Median	Quartile 3	Maximum
Intercept	−7.71E+07	−1.03E+07	−3.63E+06	−8.01E+04	7.32E+07
Population density	−2.84E+03	−2.92E+02	−2.02E+00	1.19E+02	2.91E+03
Rainfall	−7.55E+01	−2.91E+00	1.16E+01	3.06E+01	1.03E+02
Rainfall Lag 1	−1.22E+01	1.19E+01	3.03E+01	5.92E+01	1.62E+02
Rainfall Lag 2	−2.79E+01	−5.64E−01	1.30E+01	3.33E+01	1.34E+02
Rainfall Lag 3	−1.09E+02	−2.26E+01	9.02E−01	1.45E+01	7.10E+01
Average temperature	−1.02E+04	−1.62E+02	4.14E+03	1.07E+04	4.49E+04
Relative humidity	−6.24E+03	1.35E+02	1.30E+03	3.19E+03	1.11E+04
Wind speed	−3.20E+04	−9.74E+02	1.89E+03	7.39E+03	3.63E+04
Atmospheric pressure	−1.39E+05	1.69E+03	3.46E+04	1.02E+05	5.39E+05
Built area	−3.29E+04	−4.85E+01	4.76E+01	2.16E+02	1.84E+04
Crops area	−1.32E+06	−2.25E+02	−2.57E+01	2.54E+02	4.25E+06
Water area	−6.18E+05	−1.08E+04	−5.30E+02	5.22E+03	3.85E+06
Trees area	−2.16E+07	−1.90E+02	7.04E+00	2.66E+02	1.44E+06
Flooded vegetation area	−7.54E+09	−1.32E+06	8.08E+03	2.67E+06	5.10E+09
Fixed bandwidth	: 0.001				
AICc	: 30,015.01				
AIC	: 28,447.38				
BIC	: 30,962.97				
Residual sum of squares	: 43,097.96				
*R*-square	: 0.607				
Adjusted *R*-square	: 0.515				

The GWPR model explained 60.75% of dengue case variability (Adjusted *R*^2^ = 51.55%) and demonstrated robustness with an AIC of 28,447.38 and RSS of 43,097.96, indicating a strong fit for analyzing spatiotemporal determinants of dengue incidence. The significant predictors and their district-level impacts during 2017–2022 are summarized in Table 3.

**Table 3:** GWPR results with fixed exponential kernel

No	Sub-districts	Significant predictors
1	Bantul	Population Density, Average Temperature, Relative Humidity, Atmospheric Pressure, Water Area, Trees Area, Flooded Vegetation Area
2	Sewon	Population Density, Average Temperature, Relative Humidity, Atmospheric Pressure, Built Area, Crops Area, Water Area, Trees Area, Flooded Vegetation Area
3	Kasihan	Population Density, Average Temperature, Relative Humidity, Wind Speed, Built Area, Crops Area, Water Area, Trees Area, Flooded Vegetation Area
4	Sedayu	No significant predictors
5	Pajangan	Crops Area, Flooded Vegetation Area
6	Pandak	Population Density, Rainfall Lag 1, Relative Humidity, Water Area, Flooded Vegetation Area
7	Srandakan	No significant predictors
8	Sanden	No significant predictors
9	Bambanglipuro	Population Density, Rainfall, Water Area, Trees Area, Flooded Vegetation Area
10	Kretek	Built Area, Crops Area, Trees Area, Flooded Vegetation Area
11	Pundong	Population Density, Water Area
12	Jetis Bantul	Relative Humidity, Water Area, Trees Area
13	Imogiri	Rainfall Lag 1, Average Temperature, Atmospheric Pressure, Water Area, Trees Area
14	Pleret	Population Density, Rainfall Lag 1, Rainfall Lag 2, Built Area, Water Area, Trees Area, Flooded Vegetation Area
15	Banguntapan	Population Density, Rainfall Lag 1, Rainfall Lag 2, Average Temperature, Relative Humidity, Built Area, Water Area, Trees Area, Flooded Vegetation Area
16	Piyungan	Population Density, Average Temperature, Relative Humidity, Atmospheric Pressure, Crops Area, Water Area, Trees Area, Flooded Vegetation Area
17	Dlingo	No significant predictors
18	Mantrijeron	Relative Humidity, Atmospheric Pressure, Built Area
19	Kraton	No significant predictors
20	Mergangsan	Population Density, Built Area, Trees Area
21	Umbulharjo	Population Density, Rainfall Lag 2, Average Temperature, Wind Speed, Built Area, Crops Area, Trees Area
22	Kotagede	Average Temperature, Relative Humidity, Atmospheric Pressure, Built Area, Crops Area, Water Area
23	Gondokusuman	Atmospheric Pressure, Built Area, Water Area
24	Danurejan	No significant predictors
25	Pakualaman	No significant predictors
26	Gondomanan	No significant predictors
27	Ngampilan	Population Density, Built Area, Crops Area, Water Area, Trees Area, Flooded Vegetation Area
28	Wirobrajan	No significant predictors
29	Gedong Tengen	No significant predictors
30	Jetis	No significant predictors
31	Tegalrejo	No significant predictors
32	Temon	Atmospheric Pressure, Crops Area, Flooded Vegetation Area
33	Wates	Rainfall Lag 1, Rainfall Lag 3, Atmospheric Pressure, Built Area, Crops Area, Water Area, Trees Area, Flooded Vegetation Area
34	Panjatan	No significant predictors
35	Galur	No significant predictors
36	Lendah	Population Density, Built Area
37	Sentolo	Rainfall Lag 2, Trees Area
38	Pengasih	Population Density, Crops Area, Water Area
39	Kokap	Population Density, Built Area, Crops Area, Trees Area
40	Girimulyo	No significant predictors
41	Nanggulan	No significant predictors
42	Kalibawang	Water Area, Flooded Vegetation Area
43	Samigaluh	No significant predictors
44	Moyudan	Crops Area, Trees Area, Flooded Vegetation Area
45	Minggir	No significant predictors
46	Seyegan	No significant predictors
47	Godean	Population Density, Rainfall Lag 1, Rainfall Lag 3, Atmospheric Pressure
48	Gamping	Population Density, Rainfall Lag 1, Average Temperature, Relative Humidity, Wind Speed, Atmospheric Pressure, Trees Area
49	Mlati	Population Density, Water Area
50	Depok	Population Density, Rainfall Lag 1, Rainfall Lag 2, Average Temperature, Relative Humidity, Wind Speed, Atmospheric Pressure, Built Area, Crops Area, Water Area, Trees Area, Flooded Vegetation Area
51	Berbah	No significant predictors
52	Prambanan	Population Density, Average Temperature, Relative Humidity, Atmospheric Pressure, Crops Area, Water Area, Flooded Vegetation Area
53	Kalasan	Population Density
54	Ngemplak	No significant predictors
55	Ngaglik	Rainfall Lag 1, Trees Area
56	Sleman	Relative Humidity, Water Area, Flooded Vegetation Area
57	Tempel	No significant predictors
58	Turi	No significant predictors
59	Pakem	No significant predictors
60	Cangkringan	No significant predictors
61	Gedangsari	No significant predictors
62	Girisubo	No significant predictors
63	Karangmojo	Rainfall Lag 3, Relative Humidity, Built Area, Crops Area, Water Area
64	Ngawen	No significant predictors
65	Nglipar	Crops Area, Trees Area
66	Paliyan	Built Area, Crops Area, Water Area, Trees Area, Flooded Vegetation Area
67	Panggang	No significant predictors
68	Patuk	No significant predictors
69	Playen	Population Density, Rainfall Lag 2, Built Area, Crops Area, Water Area, Trees Area, Flooded Vegetation Area
70	Ponjong	Water Area
71	Purwosari	No significant predictors
72	Rongkop	No significant predictors
73	Saptosari	Population Density, Rainfall Lag 2, Rainfall Lag 3, Built Area, Crops Area, Water Area, Trees Area, Flooded Vegetation Area
74	Semanu	Population Density, Crops Area, Water Area, Trees Area
75	Semin	No significant predictors
76	Tanjungsari	No significant predictors
77	Tepus	No significant predictors
78	Wonosari	Population Density, Rainfall Lag 1, Rainfall Lag 3, Average Temperature, Relative Humidity, Wind Speed, Atmospheric Pressure, Built Area, Crops Area, Water Area, Trees Area, Flooded Vegetation Area

Table 3 highlights the localized influences of various predictors on dengue cases across sub-districts, emphasizing the need for spatially tailored intervention strategies. Sub-districts like Wonosari and Banguntapan exhibit complex dynamics requiring multidimensional approaches integrating climatic, environmental, and demographic factors, while Sedayu and Sanden show no significant predictors, suggesting unique local characteristics or unmeasured influences.

The GWPR model’s explanatory power, measured by Local R-Square values, provides critical insights into regional variations in dengue transmission, with a detailed summary of these values and their distribution presented in the supplementary table (Table S5). The distribution of Local R-Square values from the GWPR model shows significant variation in its ability to explain dengue incidence across sub-districts in the Special Region of Yogyakarta. Sub-districts with high values, such as Pakem (0.963), Cangkringan (0.960), and Girimulyo (0.941), indicate strong model performance. Moderate values are observed in Banguntapan (0.731), Moyudan (0.7291), and Wirobrajan (0.722), suggesting reasonable effectiveness with room for improvement. Lower values in Kotagede (0.275), Sedayu (0.263), and Mantrijeron (0.215) indicate that the model struggles to capture variations in these areas. Kalibawang (0.137) has the lowest value, highlighting the model’s limited explanatory power in this sub-district.

**Fig. 2:**
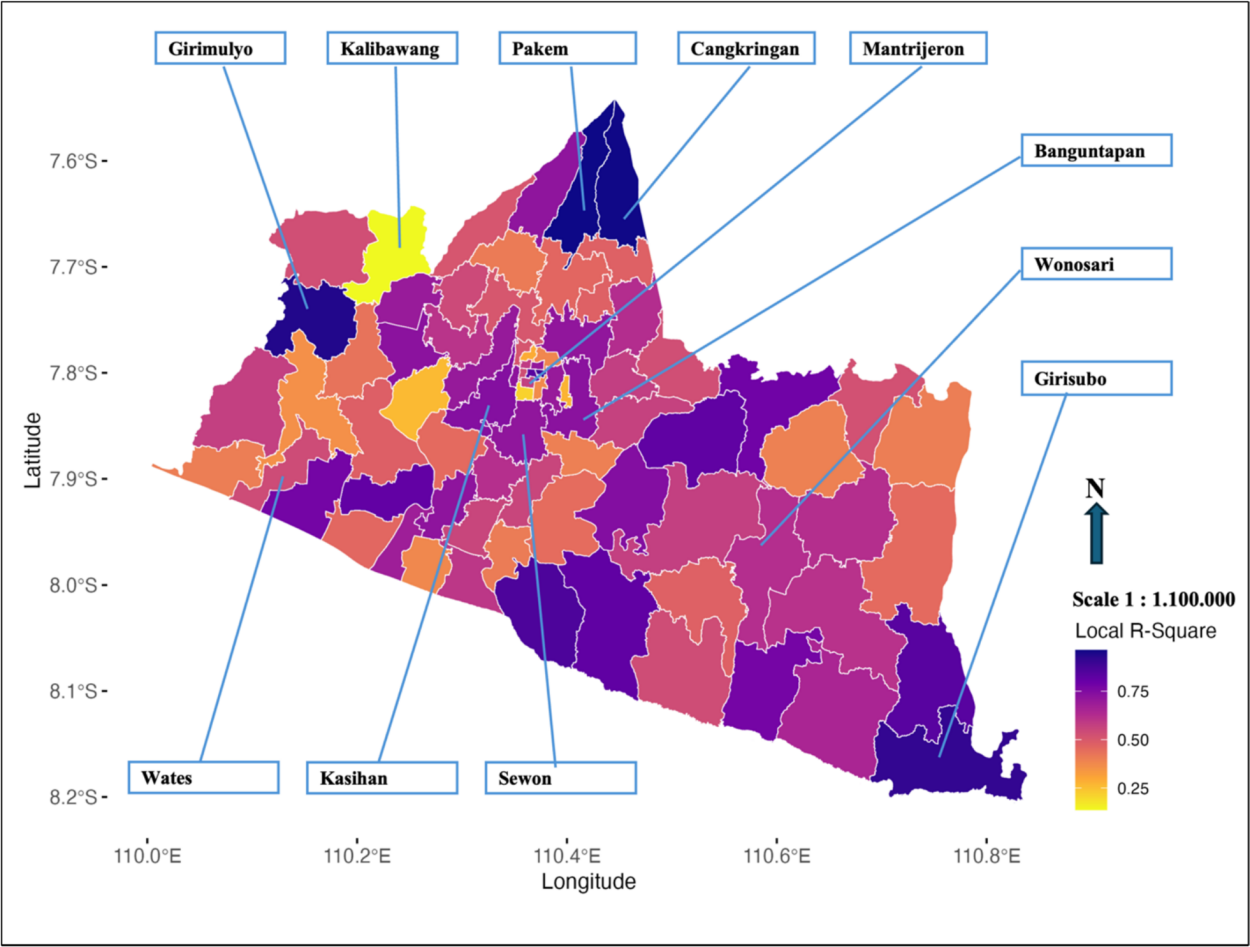
Distribution of local R-square across sub-districts based on GWPR analysis

The Local R-Square map (Fig. 2) visually represents the model’s capacity to explain dengue incidence variations, showing spatial heterogeneity in model effectiveness. Darker colors indicate better model fit, while lighter areas suggest the need for further analysis or refinement.

The Local R-Square map (Fig. 2) from the GWPR model highlights spatial heterogeneity in explaining dengue incidence across sub-districts in the Special Region of Yogyakarta. Sub-districts such as Pakem, Cangkringan, and Girimulyo exhibit high Local R-Square values (above 0.75), indicating strong model performance, while Kalibawang has very low values (below 0.25), reflecting the model’s limited explanatory power in these areas. The five sub-districts with the highest dengue cases—Kasihan (0.743), Banguntapan (0.731), Sewon (0.716), Wonosari (0.623), and Wates (0.540)—demonstrate relatively good Local R-Square values, suggesting effective model performance in high-burden areas. Key predictors in Wonosari include Rainfall Lag 1, Rainfall Lag 3, average temperature, relative humidity, wind speed, atmospheric pressure, and various land-use factors. In Wates, significant predictors include Rainfall Lag 1, Rainfall Lag 3, atmospheric pressure, and land-use factors.

The model also highlights spatial gaps in areas like Sedayu and Kalibawang, suggesting the need for additional predictors to improve explanatory power. Improvements in these low-performing areas could be achieved by incorporating missing predictors such as sanitation conditions, vector control activities, and socioeconomic factors, which may influence dengue transmission. Additionally, refining the bandwidth selection in the GWPR model could help capture local variations more effectively. Alternative modeling approaches, such as Bayesian spatial models or machine learning techniques, may further enhance predictive accuracy. Incorporating interaction terms or non-linear effects could also improve the model’s ability to capture the complex relationships between predictors and dengue incidence. The Local R-Square map further reflects the need for continuous refinement to address the spatial and temporal complexities of dengue transmission.

## Discussions

This study identified significant spatiotemporal variations in dengue incidence across the Special Region of Yogyakarta from 2017 to 2022, with 13,283 recorded cases and an average incidence of 2.37 cases per sub-district per month. The highest dengue incidence was concentrated in Kasihan, Banguntapan, and Sewon, areas characterized by high population density and accessibility, while Wonosari and Wates showed patterns influenced by climatic and land-use factors. Variations in rainfall, temperature, humidity, wind speed, and atmospheric pressure were observed across sub-districts, alongside differences in land-use characteristics, including built areas, agricultural land, tree cover, and water bodies [29, 30].

The GWPR analysis demonstrated strong spatial heterogeneity in dengue incidence, with the Fixed Exponential kernel model exhibiting high explanatory power. Sub-districts such as Pakem, Cangkringan, and Girimulyo had Local R-Square values above 0.75, indicating that dengue transmission in these areas is predominantly influenced by climatic and environmental factors [31–33], likely due to their lower population density and stable land-use patterns [13]. Conversely, areas like Kalibawang and Mantrijeron, with Local R-Square values below 0.25, suggest that additional unmeasured factors—such as sanitation, waste management, and vector control activities—may play a crucial role in dengue dynamics [34]. Urbanized sub-districts, including Banguntapan and Sewon, exhibited high dengue incidence, potentially driven by greater human-mosquito interaction, dense housing, inadequate drainage, and artificial water storage [35]. In contrast, weaker model performance in rural areas like Kalibawang may indicate the need for additional predictors related to socioeconomic disparities and healthcare access [36]. These findings emphasize the importance of integrating socio-environmental determinants into dengue risk assessments and adopting localized intervention strategies to enhance dengue prevention and control [37].

Key predictors of dengue incidence varied across sub-districts, underscoring the necessity of localized intervention strategies. In Wonosari, dengue transmission was significantly influenced by Rainfall Lag 1, Rainfall Lag 3, average temperature, relative humidity, wind speed, atmospheric pressure, and land-use factors such as built area, crops area, water area, trees area, and flooded vegetation area. Similarly, in Wates, key predictors included Rainfall Lag 1, Rainfall Lag 3, atmospheric pressure, and land-use characteristics, reinforcing the role of climate and environmental factors in shaping dengue risk [29, 32, 38, 39]. Sub-districts with complex dengue dynamics, such as Wonosari and Banguntapan, require multidimensional approaches integrating climatic, environmental, and demographic factors to address diverse transmission drivers, while regions with no significant predictors, like Sedayu and Sanden, may reflect unique local characteristics or unmeasured variables such as housing conditions, waste management, or social vulnerability [40, 41]. The role of urbanization, socioeconomic disparities, and environmental risks in shaping spatial dengue distribution has been highlighted in previous studies, reinforcing the necessity for targeted interventions [26, 37, 42, 43].

The Local R-Square mapping provides additional insights into model performance across sub-districts, highlighting areas where GWPR effectively captures dengue dynamics and regions requiring further refinement. High-performing sub-districts, such as Pakem, confirm that built areas and vegetation coverage significantly influence dengue transmission, while lower-performing areas like Sedayu and Kalibawang may require the inclusion of sanitation, healthcare accessibility, and vector control activities to improve explanatory power [38, 44]. This study reinforces the advantages of GWPR over global regression models by accounting for spatial heterogeneity and improving epidemiological modeling precision, ultimately supporting the development of spatially adaptive dengue prevention strategies [29, 34]. The findings emphasize the necessity of integrating climatic, demographic, and environmental variables into future dengue risk assessments, ensuring localized, evidence-based public health interventions for better disease control [45, 46].

To enhance dengue prevention and control, spatially adaptive interventions should be prioritized, leveraging the strengths of GWPR to identify localized risk factors and guide targeted strategies [37]. In densely populated urban areas such as Banguntapan and Sewon, vector control measures—including fogging, larval source reduction, and drainage improvements—should be intensified, especially during peak transmission periods [47, 48]. In climate-sensitive regions like Wonosari and Wates, where climatic and land-use factors influence dengue incidence, integrating land-use planning, sustainable water management, and climate-adaptive health policies is essential. Additionally, an early warning system incorporating climatic predictors could improve preparedness and response efforts [49–51]. In low-performing sub-districts like Kalibawang and Mantrijeron, sanitation, vector control measures, and socioeconomic factors may significantly influence dengue transmission. Unmeasured variables such as household waste management, drainage quality, frequency of fogging, larvicide application, accessibility to healthcare facilities, and population living conditions (e.g., housing density, access to clean water, and socioeconomic disparities) could play a crucial role in shaping local transmission dynamics. Incorporating these factors in future models may enhance predictive accuracy and inform more targeted interventions [52].

Future research should enhance GWPR applications by integrating entomological data, socioeconomic indicators, and healthcare accessibility to improve predictive accuracy, particularly in areas with unexplained transmission patterns [37, 53]. Long-term studies on seasonal variations and extreme weather events will refine temporal risk assessments [53]. Combining GWPR with machine learning and high-resolution spatial data can further advance dengue modeling, supporting precision-targeted interventions [54].

## Limitations

This study is limited by the absence of key predictors such as socioeconomic status, mobility patterns, and entomological data, which may influence dengue transmission, particularly in low-performing sub-districts like Kalibawang. The use of aggregated sub-district data may mask micro-scale variations, reducing spatial precision, especially in densely populated areas like Banguntapan and Sewon. Additionally, while climate and environmental factors were analyzed, seasonal and extreme weather influences were not fully explored, potentially affecting long-term predictions. Since findings are geographically specific to Yogyakarta, they may not be directly applicable to other regions with different environmental and socioeconomic conditions. Future studies should incorporate finer-scale data, entomological surveillance, and machine learning approaches to improve predictive accuracy and enhance targeted dengue control interventions.

## Conclusions

This study highlights the significant spatial heterogeneity in the influence of climatic, environmental, and demographic factors on dengue transmission in the Special Region of Yogyakarta. Sub-districts with high Local R-Square values, such as Wonosari, Wates, and Kasihan, demonstrate the utility of GWPR in capturing localized patterns of dengue incidence, underscoring the importance of spatially tailored intervention strategies. Conversely, low-performing sub-districts, such as Kalibawang and Sedayu, emphasize the need for additional data and predictors to refine the model and enhance explanatory power. The findings reinforce the role of spatially explicit approaches like GWPR in understanding and managing dengue dynamics, particularly in high-risk regions. Future research should integrate additional predictors and employ higher-resolution data to further enhance the effectiveness of spatial models in guiding dengue prevention and control efforts.

## Supplementary Information

Additional file 1

## Data Availability

No datasets were generated or analysed during the current study.
