# Supplementary material for: Understanding local determinants of dengue: a geographically weighted panel regression approach in Yogyakarta, Indonesia
Source: Trop Med Health. 2025 Apr 14;53:54. doi: 10.1186/s41182-025-00734-4 (PMC11995575; doi:10.1186/s41182-025-00734-4)
Supplement: Supplementary file 1 — Additional file 1 [file 41182_2025_734_MOESM1_ESM.docx]

**SUPPLEMENTARY MATERIAL**

**Table S1. Results of Multicollinearity Tests**

| **Variable** | **VIF Value** |
| --- | --- |
| Population Density | 3.099 |
| Rainfall | 2.661 |
| Rainfall Lag 1 | 2.523 |
| Rainfall Lag 2 | 2.316 |
| Rainfall Lag 3 | 1.993 |
| Average Temperature | 4.928 |
| Relative Humidity | 3.099 |
| Wind Speed | 3.378 |
| Atmospheric Pressure | 5.105 |
| Built Area | 2.011 |
| Crops Area | 1.363 |
| Water Area | 1.574 |
| Trees Area | 1.520 |
| Flooded Vegetation Area | 1.279 |

**Table S2. Results of Model Selection Tests**

| **Test** | | **Statistic** | | **P-Value** | **Conclusion** |
| --- | --- | --- | --- | --- | --- |
| Chow Test (CEM vs FEM) | 16.459 | | 1.436 E-190 | | FEM |
| Hausman Test (FEM vs REM) | 488.798 | | 2.192 E-95 | | FEM |
| Lagrange Multiplier Test (Breusch-Pagan) (REM vs CEM) | 3,993.324 | | 0,0000 | | REM |

**Table S3. Results of Classical Regression Assumption Tests**

| **Test** | **Statistic** | **P-Value** | **Conclusion** |
| --- | --- | --- | --- |
| Jarque-Bera Test (Normality) | 651,061.6 | 0.0000 | Not normally distributed |
| Breusch-Godfrey /Wooldridge Test (Autocorrelation) | 11,837.117 | 0.0000 | Autocorrelation detected |
| Breusch-Pagan Test (Heteroskedasticity) | 107.56 | 1.65E-16 | Heteroskedasticity detected |

**Table S4. Comparison and Selection of the Best GWPR Model**

| **Indicator** | **BIC** | **RSS** | **R^2^** | **R^2^adj** | **AIC** | **AICc** |
| --- | --- | --- | --- | --- | --- | --- |
| Adaptive Bisquare | 26,342.200 | 81,639.260 | 0.256 | 0.236 | 31,084.300 | 31,205.670 |
| Adaptive Gaussian | 27,040.310 | 76,145.560 | 0.306 | 0.264 | 30,819.240 | 31,083.730 |
| Adaptive Exponential | 27,075.110 | 79,276.790 | 0.278 | 0.234 | 31,023.380 | 31,261.790 |
| Fixed Bisquare | 26,778.220 | 80,487.570 | 0.267 | 0.236 | 31,064.250 | 31,251.840 |
| Fixed Gaussian | 30,509.080 | 44,318.680 | 0.596 | 0.507 | 28,533.500 | 29,959.540 |
| Fixed Exponential | 30,962.970 | 43,097.960 | 0.607 | 0.516 | 28,447.380 | 30,015.010 |

**Table S5. Local R-Square Values for Each Sub-District**

| **Sub-districts** | **R-Square** | **Sub-districts** | **R-Square** | **Sub-districts** | **R-Square** |
| --- | --- | --- | --- | --- | --- |
| Pakem | 0.963 | Minggir | 0.697 | Tempel | 0.510 |
| Cangkringan | 0.960 | Srandakan | 0.683 | Mlati | 0.508 |
| Girimulyo | 0.941 | Tepus | 0.652 | Sentolo | 0.481 |
| Girisubo | 0.914 | Kalasan | 0.625 | Paliyan | 0.478 |
| Pakualaman | 0.892 | Wonosari | 0.623 | Ngemplak | 0.478 |
| Purwosari | 0.864 | Karangmojo | 0.622 | Ngaglik | 0.463 |
| Rongkop | 0.839 | Bantul | 0.620 | Galur | 0.460 |
| Gondomanan | 0.838 | Semanu | 0.612 | Pajangan | 0.452 |
| Patuk | 0.821 | Godean | 0.608 | Ponjong | 0.452 |
| Panggang | 0.820 | Kretek | 0.600 | Imogiri | 0.447 |
| Lendah | 0.817 | Gedongtengen | 0.586 | Nanggulan | 0.431 |
| Gedangsari | 0.786 | Berbah | 0.578 | Sleman | 0.411 |
| Panjatan | 0.778 | Kokap | 0.576 | Pundong | 0.409 |
| Tanjungsari | 0.777 | Kraton | 0.576 | Semin | 0.404 |
| Dlingo | 0.746 | Playen | 0.575 | Temon | 0.404 |
| Kasihan | 0.743 | Piyungan | 0.568 | Pleret | 0.400 |
| Banguntapan | 0.731 | Bambanglipuro | 0.558 | Nglipar | 0.397 |
| Moyudan | 0.729 | Jetis Bantul | 0.558 | Gondokusuman | 0.382 |
| Wirobrajan | 0.722 | Seyegan | 0.545 | Sanden | 0.378 |
| Pandak | 0.722 | Wates | 0.540 | Mergangsan | 0.371 |
| Turi | 0.720 | Saptosari | 0.536 | Pengasih | 0.358 |
| Sewon | 0.716 | Ngampilan | 0.532 | Jetis | 0.291 |
| Depok | 0.714 | Samigaluh | 0.532 | Kotagede | 0.275 |
| Danurejan | 0.708 | Prambanan | 0.531 | Sedayu | 0.263 |
| Gamping | 0.702 | Ngawen | 0.524 | Mantrijeron | 0.215 |
| Umbulharjo | 0.699 | Tegalrejo | 0.511 | Kalibawang | 0.137 |
